# Memory on ice: spatial memory is retained over a 9-month hibernation period in Columbian ground squirrels

**DOI:** 10.1098/rsbl.2026.0220

**Published:** 2026-10-07

**Authors:** Anaïs Beauvieux, Jeremy Werner, F. Stephen Dobson, Lauriane Kuhn, Samuel Laporte, Ashley Olson, Claire Saraux, Meagan Dewar, Vincent A. Viblanc

**Affiliations:** ^1^ Future Regions Research Centre, Federation University, Australia—Berwick Campus, Berwick, Australia; ^2^ Department of Biological Sciences, Auburn University, Auburn, AL, USA; ^3^ Université de Strasbourg, CNRS, IPHC UMR 7178, F‐67000 Strasbourg, France; ^4^ ENS de Lyon, Lyon, France

**Keywords:** cognitive ecology, memory retention, torpor

## Abstract

Memory allows animals to utilize ecologically relevant information, yet whether it endures through prolonged metabolic shutdown is poorly understood. We tested spatial memory retention in a long-term, free-living hibernator, the Columbian ground squirrel (*Urocitellus columbianus*), using a field-based spatial maze over two consecutive active seasons. Ten individuals were tested before and after a 9-month hibernation period and compared with eight naive individuals. Maze-solving performances—time to exit, path sinuosity and navigational errors—were analysed over 10 consecutive trials using Bayesian nonlinear learning curves to separate retained memory from relearning. Strikingly, post-hibernation individuals with prior exposure to the task significantly outperformed naive individuals by 41–98% across all metrics, with initial performance after hibernation approaching pre-hibernation asymptotes. We show that spatial memory survives prolonged hibernation in the wild, supporting the hypothesis that neural plasticity during hibernation reflects adaptive retention rather than memory loss.

## 1. Introduction

Memory enables animals to encode, store and recall critical spatiotemporal information, such as food-resource locations [1,2] or conspecific identities [3], and is central to adaptation in complex environments [4,5]. However, cerebral function is metabolically costly [6–8] and energy availability is suggested to constrain brain evolution and cognition [9,10]. Hibernators experiencing drastic seasonal declines in energy availability, for instance, tend to have smaller brains than non-hibernators [11], and cognitive trade-offs or declines can occur during energetically demanding periods or caloric restriction [10,12–15].

Hibernation involves extended metabolic depression (metabolic rates dropping as much as 1% of normothermic levels [16]), accompanied by reduced cerebral blood flow [17], decreased brain activity [18,19] and retracted synaptic architecture and signalling [20,21]. Although such neural remodelling supports energy conservation, its consequences for memory retention remain unclear [22,23]. Some studies report post-hibernation memory impairment [24–26], whereas others do not [27–29].

We tested for long-term spatial memory retention in Columbian ground squirrel (CGS; *Urocitellus columbianus*), a champion hibernator. CGS hibernate for 8–9 months, losing >30% body mass and spending >90–95% of time in torpor [30]. During their 3–4 month active season, they rely on memory to navigate shifting territoriality and foraging resources and recall burrow systems for sheltering from predators and the elements [31,32].

Over two active seasons, 18 free-living CGS completed a spatial maze task analogous to a natural burrow system (figure 1). We measured individual performance over 10 trials: CGS retaining spatial information from previous exposures were expected to solve the maze faster and more accurately. We compared an ‘experienced’ group tested before and after hibernation with a ‘naive’ group tested only in year two. If long-term memory persists, ‘experienced’ individuals should outperform naive ones after hibernation; similar performance, indicating memory loss. Pre- and post-hibernation performance comparisons further assessed recovery of prior learning.

**Figure 1.**
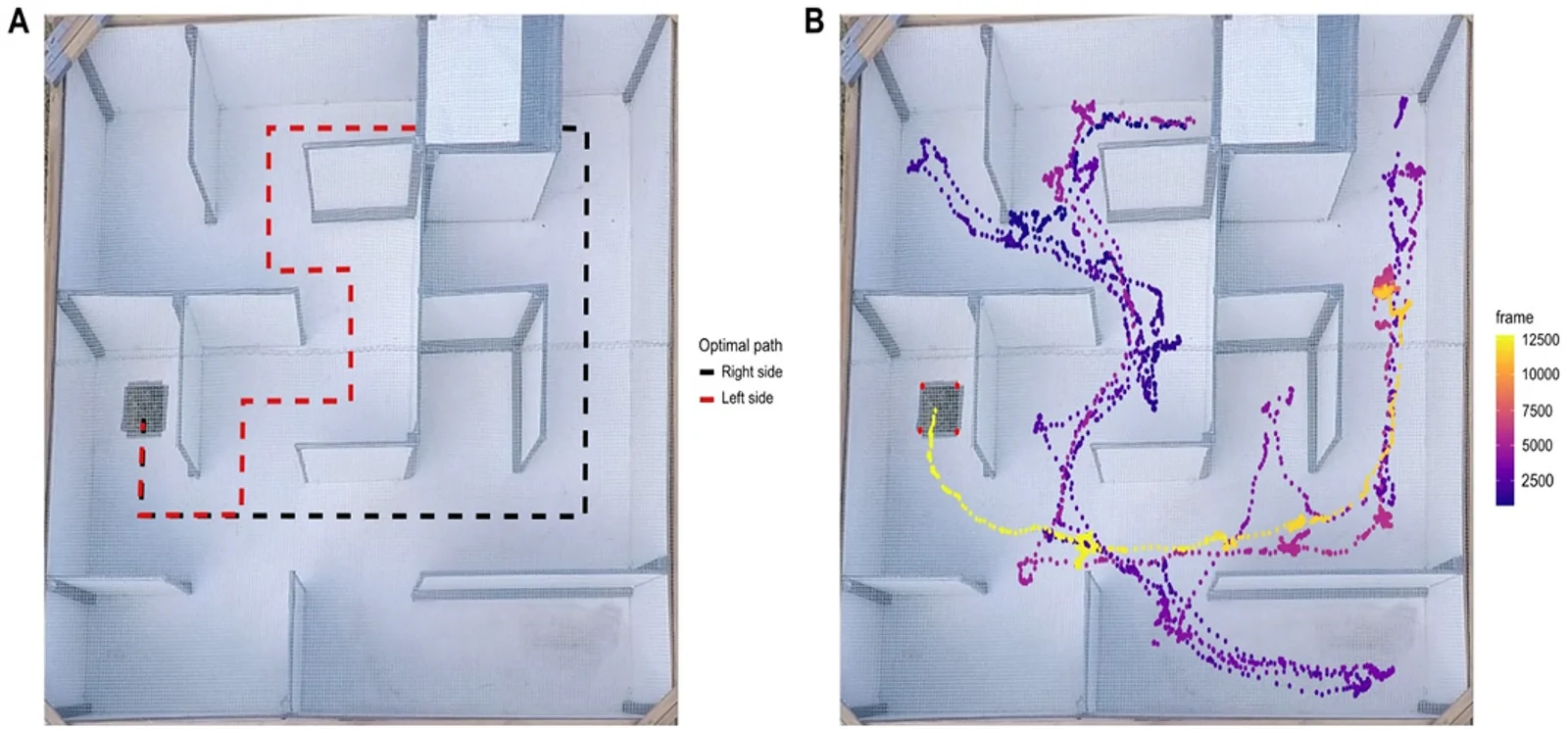
Quantifying path sinuosity: (A) maze layout with the two *a priori* optimal routes from the starting box to the exit, defined on a reference template. Optimal paths are shown for the left route (red) and the right route (black). (B) Example trial showing the animal's tracked trajectory (body centre coordinates) overlaid on the same maze image for an individual from the pre-hibernation group (trial 1, 2024). Points represent successive video frames and are coloured by frame number (early to late), illustrating the full path taken from release to exit.

## 2. Material and methods

### (a) Study animals

We studied CGS in the Sheep River Provincial Park, Alberta, Canada (50°64 N, 114°66 W, elevation 1550 m). Individuals were part of a long-term study on the ecology of CGS, and all individuals were of known age (see [33]).

In 2024, we tested 10 CGS (‘experienced’ group: 6 males and 4 females, mean age = 3.9, range = 2–10). All 10 CGS were retested in 2025 following hibernation. In 2025, we further tested eight individuals not tested in 2024 (‘naive’ group: 2 males and 6 females, mean age = 3.3, range = 2–6). Across both years, individuals were first tested on average 30.0 d (range = 28–35 d) after emergence from hibernation.

### (b) Experimental procedure

The maze (110 × 110 × 22 cm) consisted of a starting compartment, two alternative routes with branching corridors and dead ends, and a single exit opening directly on the study colony (figure 1). Solving the maze allowed individuals to return to their natural habitat and familiar burrows. The spatial location and orientation of the maze in the broader natural habitat (recorded on a 10 m × 10 m Cartesian flag grid) were identical within and across years, and the maze was positioned in a central location of the study site, prohibiting visual sensing of the tree-line or local landmarks that could have influenced orienteering [32].

All individuals were captured using Tomahawk live-traps baited with peanut butter and tested over 10 trials, once daily, mostly on consecutive days as logistically possible (mean = 10.9 d, range = 10–18). All trials were video-recorded by a GoPro Hero 7 camera, set on a perch immediately above the centre of the maze. Eighteen trials were excluded from analyses owing to issues with cameras or battery malfunction, or because the individual escaped from the maze. Therefore, 98/100 (pre-hibernation), 91/100 (post-hibernation) and 73/80 (naive group) trials were retained for analyses. Before each trial, the animal was gently placed in the dark starting compartment for 5 minutes to acclimate. The time between capture and placement in the starting box averaged 31 minutes and was not significantly correlated with performance metrics (log time to exit: *r* = –0.071, *p* = 0.254; navigational errors: *r* = –0.068, *p* = 0.278; sinuosity: *r* = –0.034, *p* = 0.594). The trial began when the starting door was remotely opened by an experimenter positioned 15 m away and ended with the individual exiting the maze. The progress of the trial was monitored in real time by the experimenter on a tablet connected over Wi-Fi to the camera. If an individual failed to exit the maze within 30 minutes, the trial was stopped and latency set to its maximum value (1800 seconds). The animal was free to move in either direction within the starting compartment and unconstrained in its choice of route in the maze. To standardize seasonal timing across individuals, all trials occurred between 13 May and 23 June in both years. The maze was cleaned between each trial with soapy water followed by 70% ethanol to remove potential scent cues [34].

### (c) Solving performance and learning metrics

Spatial memory was assessed by quantifying task performance through time to exit (seconds), path sinuosity and navigational errors (number of entries into dead ends). These three metrics captured distinct components of maze-solving performance (speed, trajectory efficiency and accuracy), allowing assessment of the consistency of memory retention across multiple performance dimensions.

‘Time to exit’ was the latency between the opening of the starting-box door and the moment the individual exited the maze. ‘Navigational errors’ were estimated as the number of entries into dead ends during a trial. ‘Path sinuosity’ was reconstructed from video recordings using markerless pose estimation (DeepLabCut^TM^ v2.2 [35]). The movement trajectory of each individual through the maze was reconstructed by tracking the position of key body parts across video frames. All videos were first standardized (cropped, rotated and trimmed), ensuring consistency in frame dimension and orientation. A deep-learning model was then trained by extracting 100 frames from each of eight representative videos (800 frames total) using *k*-means clustering to maximize pose and visual diversity. Key body parts (nose, body centre and tail base) were manually annotated to create the initial training dataset. A ResNet-50 backbone architecture was used, and training proceeded for 136 epochs (approx. 30 000 iterations; batch size = 8) until model loss plateaued. Model performance was cross-validated on videos excluded from the training data and iteratively improved by extracting and re-labelling outlier frames (low likelihood predictions or abrupt positional jumps), followed by retraining. This refinement process was repeated twice, ensuring stable predictions across individuals and trials.

For each frame, DeepLabCut^TM^ provided *X*–*Y* coordinates and a likelihood score for each body part. Predictions with likelihood values <0.7 were discarded to minimize spurious detections. Resulting trajectories were visually inspected and cleaned to remove obvious tracking artefacts. After these filtering steps, an average of approximately 70% of frames per trial were retained for subsequent analyses. Movement trajectories were reconstructed using the body-centre coordinates: the most stable and representative estimate of whole-body displacement. Total distance travelled was calculated as the sum of Euclidean distances between consecutive valid detections and expressed in cm (figure 1B). Pixel-to-cm conversion was achieved using the four fixed corners of the maze exit as spatial reference points, with median coordinates used to compute a similarity transformation correcting for translation, rotation and scale relative to a template.

Sinuosity was defined as the ratio between total distance travelled and the length of the corresponding optimal path. Optimal paths (left and right corridor-centre polylines) were defined *a priori* on a template layout and projected onto each video using the same transformation (figure 1A), with the relevant path selected according to the starting side chosen by the animal at each trial. Sinuosity values > 1 indicate deviations from the shortest possible route. Trials with insufficient detections (*n* = 9) to produce a reliable trajectory were excluded from sinuosity analyses. In cases where missing early detections resulted in a reconstructed trajectory shorter than the optimal path, total distance was conservatively set to the optimal path length (sinuosity = 1).

All procedures carried out were subject to IACUC approval by Auburn University and the IPHC. Authorization for conducting research and collecting samples in the Sheep River Provincial Park was obtained from Alberta Environment and Parks and Alberta Fish & Wildlife (research permits 24-192, 25-068).

### (d) Data analyses

#### (i) Learning-curve modelling

We modelled task performance (time to exit, path sinuosity and navigational errors) across trials using a nonlinear learning-curve approach. This approach, preferred over linear or breakpoint analyses, yielded three biologically interpretable parameters: initial performance (hereafter *start*: value of the parameter at the first trial), asymptotic performance (*asymptote*: the performance level approached as trials increased) and learning rate (*k*: the rate at which performance approached the asymptote). Separate models were fitted for each metric: time to exit and path sinuosity were log-transformed before analysis, and navigational errors were modelled using a negative binomial distribution (log-link) to account for over-dispersion in count data.

We used Bayesian nonlinear mixed models to obtain reliable parameter estimation for learning-curve models with random effects under limited sample sizes, allowing straightforward inference on biologically meaningful contrasts using posterior draws. Weakly informative priors were used to provide mild regularization. For *start* and *asymptote*, normal priors centred on the mean of the response variable (on the log-scale), with a standard deviation of twice the observed standard deviation. This anchored estimates to the scale of the data without strongly constraining posterior values. For *k* and residual variance parameters, exponential priors with a lower bound of zero were used, reflecting the constraint that these parameters must be strictly positive. The three parameters (*start*, *asymptote* and *k*) were allowed to vary among groups (‘experienced’ animals before and after hibernation, and ‘naive’ animals) and individual identity was included as a random effect to account for repeated measures and consistent among-individual differences in baseline performance. Age (z-scored), sex and maze route side (left versus right) were included as covariates to control for baseline variation unrelated to memory retention. One individual in the pre-hibernation group and one in the naive group did not exit the starting box on their first trial; route side could therefore not be determined for these individuals, resulting in slightly smaller sample sizes (pre-hibernation: *n* = 97; post-hibernation: *n* = 91; naive: *n* = 72).

Models were fitted using four Markov chains of 4000 iterations each, with a warmup of 2000 iterations per chain, yielding 8000 post-warmup posterior draws in total. Model convergence was assessed using standard diagnostics ($\hat{R}$ < 1.01 and ESS > 1000 for all parameters).

#### (ii) Memory retention and savings

Retention of spatial information across hibernation was assessed by comparing the start parameter between ‘experienced’ and ‘naive’ groups tested in the same year. Lower start values in ‘experienced’ animals would indicate retention of the previously acquired spatial task, whereas similar values between groups would support a memory reset. To evaluate performance changes over hibernation, we compared start, asymptote and *k* values for ‘experienced’ animals before and after hibernation. Similar parameter values would indicate stable performance, whereas higher *start*, lower *k*, or reduced *asymptote* after hibernation would suggest performance degradation, reflecting impaired initial performance, slower learning, or reduced performance capacity.

We also compared learning savings (accelerated rate of reacquisition of the task owing to prior experience) by comparing *k* between ‘experienced’ animals after hibernation and ‘naive’ individuals. Inference was based on pairwise contrasts between groups derived from posterior draws, summarized using 95% credible intervals (CI) and posterior probabilities that contrasts were in the predicted direction. Posterior probabilities of 1.00 indicate that all 8000 posterior draws were in the predicted direction.

All analyses were conducted in R [36]. Bayesian nonlinear mixed models were fitted using *brms* [37] with Stan via *rstan* [38]. Pairwise contrasts between groups were computed using the *posterior* package [39].

## 3. Results

### (a) Maze completion duration

Maze completion duration decreased across trials in all groups (figure 2A). Following hibernation, ‘experienced’ individuals exited the maze roughly 44 times (approx. 17 seconds) faster at first trial than ‘naive’ individuals (approx. 736 seconds; difference in start on the log-seconds scale: mean = –3.83; 95% CI, –5.35 to –2.51; *P*(Δ < 0) = 1.00). ‘Experienced’ individuals also substantially improved their performance compared with their first trial in the pre-hibernation session (approx. 243 seconds), exiting the maze roughly 14 times faster (Δstart = –2.70; 95% CI, –3.44 to –2.01; *P*[Δ < 0] = 1.00). Overall, their initial post-hibernation performance was similar to their pre-hibernation asymptote (Δstart–asymptote = 0.45; 95% CI, –0.99 to 1.72; *P*[Δ > 0] = 0.76).

**Figure 2.**
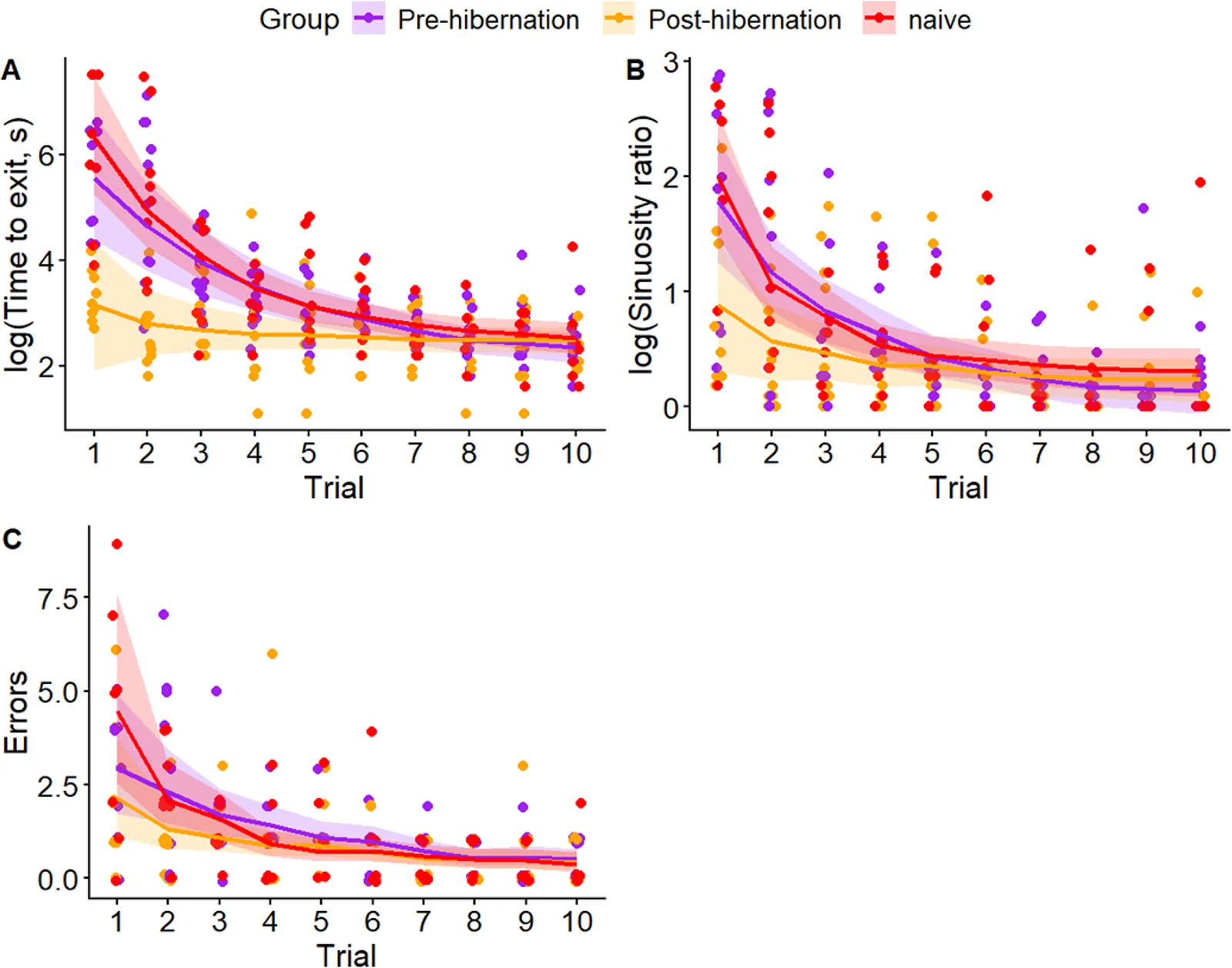
Learning curves for (A) log-transformed time to exit the maze, (B) log-transformed sinuosity ratio (trajectory length relative to the optimal path), and (C) number of errors across 10 trials. Points represent trial-level observations for ‘experienced’ individuals before (pre, *n* = 97 trials) and after (post, *n* = 91 trials) hibernation, tested in 2024 and 2025, and ‘naive’ individuals (*n* = 72 trials, tested only in 2025). Solid lines show marginal population-level predictions from Bayesian nonlinear mixed learning-curve models (exponential approach to an asymptote), averaged over the observed age (standardized), sex and route side distributions within each group. Ribbons indicate 95% credible intervals.

We found no strong evidence that learning rates differed between ‘naive’ individuals and ‘experienced’ individuals following hibernation (Δ*k* = 0.43; 95% CI, –0.16 to 2.12; *P*[Δ*k* > 0] = 0.81). Initial performance was positively, albeit weakly, affected by age (0.68; 95% CI, 0.05–1.38), but not by sex (0.46; 95% CI, –0.69 to 1.80). Maze route side had a negligible effect on initial performance (–0.10; 95% CI, –0.61 to 0.43).

### (b) Trajectory sinuosity

Sinuosity decreased across trials in all groups (figure 2B). ‘Experienced’ individuals exhibited 1.7 times lower sinuosity at their first trial after hibernation than ‘naive’ individuals (difference in start: mean = –0.54; 95% CI, –1.28 to –0.13; *P*[Δ < 0] = 0.94). For ‘experienced’ individuals, initial sinuosity was also 1.5 times lower after hibernation than before, at first trial (mean = –0.45; 95% CI, –0.93 to –0.02; *P*[Δ < 0] = 0.97). However, initial post-hibernation sinuosity remained higher than the pre-hibernation asymptote (Δstart–asymptote = 1.81; 95% CI, 1.06–2.48; *P*[Δ > 0] = 1.00).

There was no strong evidence that learning rates differed between ‘naive’ individuals and ‘experienced’ individuals following hibernation (Δ*k* = 0.04; 95% CI, −0.46 to 1.19; *P*[Δ*k* > 0] = 0.43). Neither age (–0.03; 95% CI, −0.31 to 0.24) nor sex (−0.55; 95% CI, −1.10 to 0.11) significantly affected initial sinuosity. Maze route side significantly affected initial sinuosity even after normalization to each route's optimal path. Animals taking the right route showed lower sinuosity, on average (–1.02; 95% CI, –1.46 to –0.57; *P*[Δ < 0] = 1.00).

### (c) Navigational errors

Error counts declined across trials in all groups (figure 2C). ‘Experienced’ individuals made approximately 1.7 times fewer errors at their first trial after hibernation than ‘naive’ individuals at the same time (difference in start on the log-mean scale: mean = –0.53; 95% CI, –1.22 to 0.16; *P*[Δ < 0] = 0.94). For ‘experienced’ animals, initial error counts did not differ significantly before and after hibernation (mean = –0.17; 95% CI, –0.76 to 0.41; *P*[Δ < 0] = 0.72). By contrast, initial post-hibernation error counts were higher than the pre-hibernation asymptote (Δstart–asymptote = 2.85; 95% CI, 1.42 to 4.56; *P*[Δ > 0] = 1.00).

Finally, learning rates did not differ significantly between ‘naive’ individuals and ‘experienced’ individuals following hibernation (Δ*k* = –0.04; 95% CI, –0.41 to 0.34; *P*[Δ*k* > 0] = 0.35). Neither age (0.02; 95% CI, –0.20 to 0.25) nor sex (–0.46; 95% CI, –0.92 to 0.01) significantly affected initial error rates. Maze route side also significantly affected initial error counts. Animals taking the right route made fewer errors, on average (–0.69; 95% CI, –1.19 to –0.21; *P*[Δ < 0] = 0.99).

## 4. Discussion

Our results show that CGSs retained spatial memory over 9 months of hibernation, providing empirical evidence that memory retention can occur over prolonged periods of metabolic depression despite substantial neural remodelling [23]. ‘Experienced’ individuals outperformed ‘naive’ ones by 41–98% in maze completion duration, trajectory sinuosity and navigational errors from the first post-hibernation trial. They also improved in completion duration (approx. 93% faster) and sinuosity (approx. 36% lower), compared with their pre-hibernation exposure, but not in initial error counts. Post-hibernation learning rates were similar between groups, probably because ‘experienced’ CGS retained high performance, close to pre-hibernation levels. ‘Naive’ individuals required at least five trials to match the initial performance of ‘experienced’ ones, highlighting the advantage of retained spatial information. This aligns with previous findings in captive golden hamsters [27], emphasizing the importance of memory retention for re-actualization rather than re-learning of previously acquired spatial tasks.

Understanding spatial memory persistence during hibernation involves considering the neurobiological mechanisms protecting memory storage. The hippocampus plays a central role at the interface of spatial navigation and episodic memory formation [40,41], with place cells in CA1 and CA3 involved in spatial encoding and memory consolidation [42–44]. Neuronal spine regressions in CA3 during hibernation in hamsters did not impair hippocampal-dependent memory [27], suggesting neural retractions may not cause memory loss. Our results support this, aligning with the idea that neural plasticity during hibernation reflects adaptive reorganization [20,45,46].

However, some species show reduced post-winter memory retention following hibernation, including bats [24] and European ground squirrels [25]. In grey mouse lemurs, reduced retention was observed in both hibernating females and non-hibernating males, suggesting that factors other than hibernation drive the observed memory decline [47]. Hypometabolic states may affect memory retrieval rather than storage *per se* [48]. Because the formation and maintenance of dendritic spines is integral to information processing and memory engrams [49], species- and individual-specific variation in spine parameters and neural regrowth following hibernation might explain differences in memory retrieval. We tested squirrels several weeks after hibernation, when neural regrowth would already have occurred. Testing immediately upon emergence might help to distinguish between recall and storage hypotheses.

In our maze, squirrels navigated interconnected corridors with dead ends to find an unobstructed exit. In natural burrow systems, openings shift dynamically over and within seasons, as they are built, maintained, or abandoned by individuals (A.B., V.A.V., C.S., F.S.D., personal observations). Selection on spatial memory and recall mechanisms should thus be critical in this prey species, allowing rapid re-actualizing and updating of cognitive maps, including escape routes, of the landscape [31,32,50]. Future studies should explore the speed of spatial updating post-hibernation, and whether retention extends to other types of memory, like social memory (e.g. [51]).

## Data Availability

All data and R code required to reproduce the analyses and figures presented in this article are available from the data.InDoRES repository [52].
